# Analysis of Different Hyperspectral Variables for Diagnosing Leaf Nitrogen Accumulation in Wheat

**DOI:** 10.3389/fpls.2018.00674

**Published:** 2018-05-23

**Authors:** Changwei Tan, Ying Du, Jian Zhou, Dunliang Wang, Ming Luo, Yongjian Zhang, Wenshan Guo

**Affiliations:** Jiangsu Key Laboratory of Crop Genetics and Physiology, Co-Innovation Center for Modern Production Technology of Grain Crops, Joint International Research Laboratory of Agriculture and Agri-Product Safety of the Ministry of Education of China, Yangzhou University, Yangzhou, China

**Keywords:** wheat, leaf nitrogen accumulation, hyperspectral remote sensing, vegetation index, diagnostic model

## Abstract

Hyperspectral remote sensing is a rapid non-destructive method for diagnosing nitrogen status in wheat crops. In this study, a quantitative correlation was associated with following parameters: leaf nitrogen accumulation (LNA), raw hyperspectral reflectance, first-order differential hyperspectra, and hyperspectral characteristics of wheat. In this study, integrated linear regression of LNA was obtained with raw hyperspectral reflectance (measurement wavelength = 790.4 nm). Furthermore, an exponential regression of LNA was obtained with first-order differential hyperspectra (measurement wavelength = 831.7 nm). Coefficients (*R*^2^) were 0.813 and 0.847; root mean squared errors (RMSE) were 2.02 g·m^−2^ and 1.72 g·m^−2^; and relative errors (RE) were 25.97% and 20.85%, respectively. Both the techniques were considered as optimal in the diagnoses of wheat LNA. Nevertheless, the better one was the new normalized variable *(SD*_*r*_ − *SD*_*b*_*)/(SD*_*r*_ + *SD*_*b*_*)*, which was based on vegetation indices of R^2^ = 0.935, RMSE = 0.98, and RE = 11.25%. In addition, *(SD*_*r*_ − *SD*_*b*_*)/(SD*_*r*_ + *SD*_*b*_*)* was reliable in the application of a different cultivar or even wheat grown elsewhere. This indicated a superior fit and better performance for *(SD*_*r*_ − *SD*_*b*_*)/(SD*_*r*_ + *SD*_*b*_*)*. For diagnosing LNA in wheat, the newly normalized variable *(SD*_*r*_ − *SD*_*b*_*)/(SD*_*r*_ + *SD*_*b*_*)* was more effective than the previously reported data of raw hyperspectral reflectance, first-order differential hyperspectra, and red-edge parameters.

## Introduction

Nitrogen fertilizers are currently used to produce crops of high yield and good quality. Nitrogen concentration is the focus of attention in plant nutrition, which is determined by the characteristics of nitrogenous fertilizers. Nitrogen fertilizers are preferred as they are high permeable in soil. Nitrogen fertilizers must be applied to the soil at regular intervals to provide nitrogen nutrition. The basis of variable fertilization is based on the following principle: the dynamics of nitrogen absorption, growth, and nutritional requirements of crops. It is necessary to accurately determine the amount of nitrogen fertilizers, which is required for the growth and proliferation of plants.

Leaf nitrogen accumulation (LNA) is defined as the product of leaf nitrogen content (LNC) and leaf dry weight. It not only reflects the individual information of LNC, but also includes group characteristics of vegetation coverage. The level of LNA would improve crop nitrogen nutrition, which increases LNC and vegetation coverage. Nitrogen fertilizers are theoretically significant for crop growth and nitrogen diagnosis. Moreover, it is essential to investigate the quantitative relationship between LNA and leaf spectrum. This reflects the integrated characteristics of crop group and leaf spectrum. Chemical analysis of plant tissues must be performed to diagnose LNA of wheat. It is a time-consuming and laborious process. Remote sensing was performed accurately and quickly with the following objectives: diagnosis of crop nutrient status, monitoring of crop growth, and evaluation of crop yield (Migdall et al., 2009; Piekarczyk et al., 2011; Teke et al., 2013). Nitrogen status of crops was determined by establishing a hyperspectral diagnostic model for the nitrogen crop accumulation. Practically, this information was significant for effective nitrogen fertilization (Jain et al., 2007; Mahajan et al., 2014; Morier et al., 2015). Several studies have been conducted on hyperspectral remote sensing and inversion of crop components. Some simulation models, which are feasible and widely applicable in various industries, have also been established till date (Takahashi et al., 2000; Chanseok et al., 2009; Shwetank et al., 2010; Onoyama et al., 2015; Marshall et al., 2016). Currently, R_780_/R_740_ was considered as an optimal ratio index that established the uptake of nitrogen in maize above the ground (Mistele and Schmidhalter, 2008, 2010). Moreover, R_760_/R_730_ was used to accurately predict aboveground nitrogen accumulation in wheat (Erdle et al., 2012). Horler et al. (1983) found a significant correlation between “red edge” and vegetation chlorophyll concentration. This implies that it was possible to calculate vegetation chlorophyll concentration with red-edge parameters of remote sensing. This indicates that the reflectivity of visible light increased due to nitrogen deficiency in plants; however, reflectance was different in different plants. Reflectance was negatively correlated with chlorophyll content and carotenoid (Thomas et al., 1987). Red edge parameter also reflects nitrogen status of crops. Tarpley et al. (2000) proposed that a combination of red edge wavelengths must be selected from very near infrared wavelengths to accurately and precisely predict LNA in cotton. Visible and red-edge bands were used to assess nitrogen changes in crops; these changes were observed in accordance with hyperspectral features of chlorophyll (Chen et al., 2010; Feng et al., 2014). Shibayama et al. (1993) conducted a study on wheat crops and found a good regression relationship between LNA per unit area and a linear combination of hyperspectral reflectance at 620 and 760 nm. A linear combination of hyperspectral reflectance was observed at 400 and 880 nm, respectively. Wessman et al. (1988) investigated chemical composition of wheat crown with first and second derivative of forest spectra. Optimal spectra combination showed the strongest correlation with biomass. Continuum removal analysis, stepwise multiple linear regression (SMLR), and partial least squares (PLS) were the methods used to obtain hyperspectral data, which was then used to determine concentrations of biochemical constituents in plants with hyperspectral data (Lacapra et al., 1996; Martin and Aber, 1997; Gupta et al., 2003; Hatfield et al., 2008; Ecarnot et al., 2013). Many researchers focused on investigating the relationships between vegetation indices and nitrogen accumulation of plants. Zhu et al. (2008) found that in both wheat and rice, LNA could be determined with common vegetation indices (VIs); moreover, LNA can be monitored effectively with separate regression equations. Moreover, RVI (870, 660) and RVI (810, 660) showed a high correlation to LNA in both wheat and rice crops. In addition, the relationship between VIs and LNA was far more focused than the relationships between single wavebands and LNA in both wheat and rice. Several studies have described how the nutritional value of wheat can be monitored with remote sensing; however, very few studies have described how remote sensing parameters, such as raw hyperspectral reflectance, first-order differential hyperspectra, and remote sensing vegetation index, can be comprehensively used to investigate LNA of wheat.

In the present study, researchers investigated the relationship between wheat LNA and various hyperspectral remote sensing parameters. Furthermore, hyperspectral characteristics and sensitive analysis were performed to identify LNA in wheat. Moreover, researchers also determined the quantitative relationships associated with LNA in wheat. The expected results were used to provide a technical approach to non-destructive monitoring and diagnosis of nitrogen status in crops.

## Materials and methods

### Experimental design

Experiments were conducted in the experimental field of Agricultural College of Yangzhou University from 2015 to 2016 (119°23′26″E, 32°23′53″N), and in the experimental farming of Yangzhou University in 2017 (119°26′22″E, 32°25′34″N). Representative wheat cultivars were Yangmai 6, Yangmai 16, and Ningmai 9 in 2015, 2016, and 2017, respectively. The former crop was rice, while sandy loam was the soil texture. In the soil layer of 0–30 cm, soil organic matter was 21.1 g.kg^−1^ and available nitrogen was 104.2 mg; moreover, available phosphorus was 25.3 mg and available potassium was 87.1 mg. To investigate differences in the growth and biochemical composition of wheat, five nitrogen levels were defined in this experiment: N_0_ (no nitrogen fertilizer), N_1_ (7.5 g N·m^−2^), N_2_ (15 g N·m^−2^), N_3_ (22.5 g N·m^−2^), and N_4_ (30 g N·m^−2^). Each level was subjected to three repeats. The plot area was 15 × 10 m; test crops were distributed randomly in the field. Conventional methods of cultivation were used in this experiment. The 2016 data was used as a training set, while the 2015 and 2017 data was used as a test set, respectively.

### Determination of hyperspectral reflectance

Hyperspectral reflectance was determined with Unispec spectrometer (PPS Scientific Instruments, Inc., UK). The instrument has a built-in light source (7.0 W halogen bulb). A 50 W halogen lamp was placed besides the instrument to provide auxiliary lighting. A light source, which had an azimuthal inclination of 70°, was placed 45 cm away from the surface of the sample. The working temperature of the instrument was in the range 0–45°C; wavelength range was 300–1,100 nm, and viewing angle was set at 8°. Hyperspectral resolution was less than 10 nm when the wavelength was in the range 300–1,100 nm. Hyperspectral resolution was less than 7 nm in the wavelength range of 400–730 nm. Hyperspectral resolutions were determined with an accuracy of 0.3 nm and 0.2 nm, respectively. The scan time was less than 0.5 s.

The five key growth stages of wheat were classified as follows: seedling stage, jointing stage, heading stage, flowering stage and grain filling stage. At each of the five stages of wheat cultivation, samples were collected on a day when the weather was bright and sunny. Between 10:00 a.m. and 12:00 p.m., three to five plants were sampled to determine the growth level of wheat with respect to nitrogen levels. To determine hyperspectral reflectance of wheat leaves, samples were packed in plastic bags and kept in the refrigerator of a laboratory. Hyperspectral reflectance was determined approximately 2 cm above leaves and headed down vertically. To eliminate the effect of shadow, leaves were covered with black cloth that exhibited an almost zero reflectivity. Three to five fully developed leaves were removed from the top, and six representative points were selected from the surface of each leaf. Each point was repeated ten times, and the average was considered as hyperspectral reflectance. A BaSO_4_ reference plate was used to account for corrections, immediately before and after the determination of each point. Equation (1) was used to determine hyperspectral reflectance:

(1) $$\begin{array}{l} R_{mi}=\frac{DN_{mi}}{DN_{ri}}\cdot R_{ri} \end{array}$$

Here, *R*_*mi*_ was the reflectance of the sample at wavelength λ_i_; *R*_*ri*_ was the reflectance of the diffuse reflectance reference plate at wavelength λ_i_; *DN*_*mi*_ was the radiance value of the sample at wavelength λ_i_; *DN*_*ri*_ was the radiance value of diffuse reflectance reference plate at wavelength λ_i_. Among them, *DN*_*mi*_ and *DN*_*ri*_ were actual measured values, whereas *R*_*ri*_ was a known calibrated value for the reference plate.

### Determination of leaf nitrogen accumulation

Twenty strains of wheat were taken at each sampling stage to synchronize hyperspectral measurements. For three to five plants in a plot, nitrogen accumulation of leaves was measured at following stages: seedling stage, jointing stage, heading stage, flowering stage, and grain filling stage of wheat. Fresh leaves of wheat were incubated at 105°C for 15 min, and they were oven-dried at 70°C until a constant weight was reached. Dried leaf samples were ground to pass through 1 mm screen. They were then stored in plastic bags for chemical analysis. Total nitrogen concentration in leaf tissues was determined by micro-Keldjahl method. Thereafter, LNC (%) was expressed on the basis of unit dry weight. Finally, LNA (g·m^−2^) was calculated as the product of LNC (%) and unit leaf dry weight (g·m^−2^).

### Hyperspectral characteristic variables

Based on previous studies, A total of 18 hyperspectral characteristic variables were considered (Table 1), which were related to LNA. Hyperspectral characteristic variables of wheat were determined in previous studies. The most common hyperspectral characteristic variables of wheat were as follows: characteristic variables extracted from raw hyperspectra and first-order differential hyperspectra, and characteristic variables based on hyperspectral positions, hyperspectral area and hyperspectral vegetation indices.

**Table 1:** Definitions of hyperspectral remote sensing parameters used in the study.

	**Variable**	**Definition**	**Description**	**References**
Hyperspectral characteristic variables based on red-edge position	*D*_r_	First-order maximal derivative inside red edge	Red edge covers 680-780 nm; *D*_r_ is the maximum of first-order differential spectra within red edge	Gong et al., 2002
	λ_r_	Red edge position	λ_r_ is the wavelength with respect to *D*_r_ (nm)	
	*D*_b_	First-order maximal derivative inside blue edge	Blue edge covers 490–530 nm; *D*_b_ is the maximum of first-order differential spectra within blue edge	
	λ_b_	Corresponding band length with *D*_b_	λ_b_ is the wavelength with respect to *D*_b_ (nm)	
	*D*_y_	First-order maximal derivative inside yellow edge	Yellow edge covers 550–582 nm; *D*_y_ is the maximum of first-order differential spectra within yellow edge	
	λ_y_	Corresponding band length with *D*_y_	λ_y_ is the wavelength with respect to *D*_y_ (nm)	
	*R*_g_	Hyperspectral reflectance at the peak of green band	*R*_g_ is the maximal reflectance for the wavelength of 510–560 nm	
	λ_g_	Corresponding band length with *R*_g_	λ_g_ is the wavelength with respect to *R*_g_ (nm)	
	*R*_o_	Hyperspectral reflectance at valley of red band	*R*_o_ is the maximal reflectance for the wavelength of 640-680 nm	
Hyperspectral characteristic variables based on red-edge area	*SD*_r_	Summation of first-order derivatives inside red edge	The sum of the values of first-order differential spectra in the red edge wavelength range	
	*SD*_b_	Summation of first-order derivatives inside blue edge	The sum of the values of first-order differential spectra in the blue edge wavelength range	
	*SD*_y_	Summation of first-order derivatives inside yellow edge	The sum of the values of first-order differential spectra in the yellow edge wavelength range	
Hyperspectral characteristic variables based on vegetation indices	*SD*_r_*/SD*_b_	–		
	*SD*_r_*/SD*_y_	–		
	*R*_g_*/R*_o_	–		
	*(SD*_r_*−SD*_b_*)/(SD*_r_*+SD*_b_*)*	–		
	*(SD*_r_*−SD*_y_*)/(SD*_r_*+SD*_y_*)*	–		
	*(R*_g_*−R*_o_*)/(R*_g_*+R*_o_*)*	–		

### Data analysis

The means of all nitrogen treatments were compared to determine changes in LNA and hyperspectra of different organs. To develop regression models, data was collected from 144 samples in 2016. To evaluate regression models, data was obtained from field experiments of 150 samples in 2015 and 120 samples in 2017, respectively. To establish the relationship between LNA and 18 hyperspectral characteristic variables, of the following regression models were used: linear, exponential, logarithmic, parabolic, power, and cubic. Based on statistical significance (*p* < 0.05 or 0.01), models were ranked as correlation coefficients (r in case of linear models) and coefficients of determination (*R*^2^ in case of non-linear models). Differential transformation of reflectance spectrum was carried out to accommodate an over-fitting phenomenon. The established model was verified with measured sample value, and an over-fitting phenomenon was mitigated. Linear and non-linear correlation analyses were performed for the following parameters: raw hyperspectra, first-order differential hyperspectra, and LNA. In the key spectrum, sensitive hyperspectral variables were related to LNA. Using a self-developed computer program based on MATLAB 7.0 software (The MathWorks, Inc., Natick, MA, USA), 18 hyperspectral characteristic variables (Table 1) were calculated from raw data of hyperspectral reflectance. Then, linear, exponential, logarithm, parabolic, or power correlation analyses were used to determine 18 hyperspectral characteristic variables and LNA. Thus, we identified hyperspectral characteristic variables associated with LNA. Then, the best-fit *R*^2^ model was determined from linear or non-linear models. The relationship between LNA and hyperspectral characteristic variables was determined using SPSS18.0 software. A 1:1 scale was used to determine the estimated and measured LNA values. To estimate LNA and determine the performance of the model, we used following parameters: *R*^2^, root mean squared error (RMSE), and relative error (RE). Higher the value of *R*^2^, lower would be RMSE and RE values and higher would be the accuracy of estimating LNA. Equations (2, 3) were used to calculate RMSE and RE, respectively:

(2) $$\begin{array}{l} RMSE=\sqrt{\frac{1}{n}\overset{n}{\underset{i=1}{\sum }}{\left(y_{i}-{ŷ}_{i}\right)}^{2}} \end{array}$$

(3) $$\begin{array}{l} \text{RE}\left(\text{\%}\right)=\left({\text{y}}_{\text{i}}-{\hat{\text{y}}}_{\text{i}}\right)/{\text{y}}_{\text{i}}\times 100 \end{array}$$

Here, *y*_*i*_ and ŷ_*i*_ represented measured and predicted values of wheat LNA, respectively; *n* denoted the number of samples.

### Distribution of LNA

The data of 2016 was used as a training set, while the data of 2015 was used as a test set. Table 2 showed that samples of both training and test sets had similar values for following parameters: amplitude, average, standard deviation, and standard error. Moreover, training and test sets were independent of each other. Thus, a reliable model was established and validated by using these data sets.

**Table 2:** Distribution of LNA in training and test datasets.

**Sample set**	**Year**	**Sample size**	**Maximum value (g m^−2^)**	**Minimum value (g m^−2^)**	**Average value (g m^−2^)**	**Standard deviation (g m^−2^)**	**Standard error (g m^−2^)**
Training set	2016	144	17.556	1.52	7.858	3.742	0.306
Test set	2015	150	18.835	0.312	8.024	4.617	0.378

### Correlation analysis and diagnostic model of wheat LNA with raw hyperspectral reflectance

**Figure 1:**
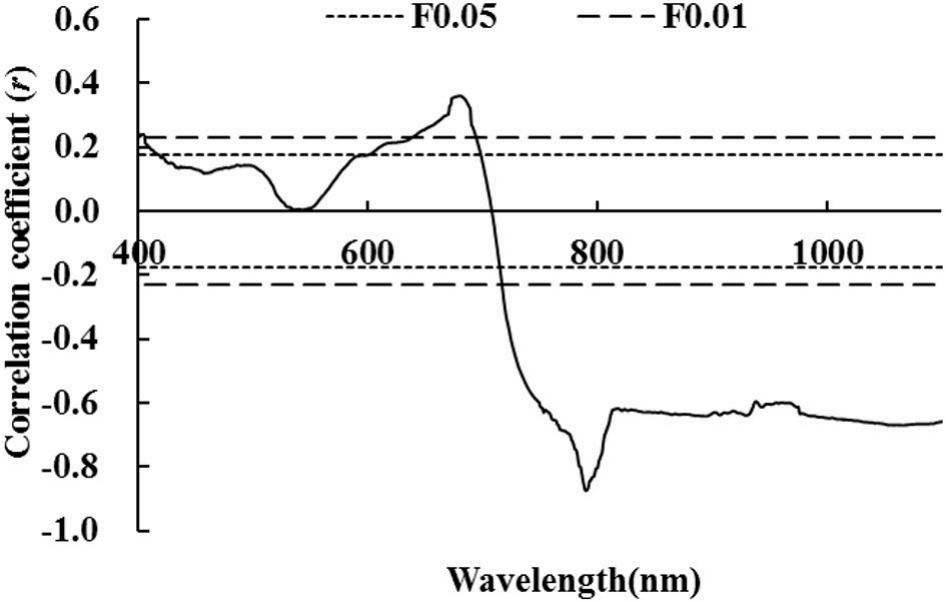
Correlation coefficient between raw hyperspectral reflectance and wheat LNA (The correlation coefficient outside the two P_0.05_ lines was significant at 0.05 level, while the correlation coefficient outside the two P_0.01_ lines was significant at 0.01 level).

As shown in Figure 1, regression relationships of LNA were established with respect to raw hyperspectral reflectance in wheat. In the visible light range of 420–600 nm, there was no significant correlation between LNA and raw hyperspectral reflectance. A significant correlation was observed between other regions of visible light spectra; however, none of them was ideal because of the number of samples and random error. Moreover, this may be due to a strong absorption of chlorophyll in the visible region. Furthermore, the interaction between chlorophyll and internal structure of leaves was investigated. Absolute values of r were always greater than 0.25 in the near-infrared region of the spectra. This indicates that absolute values of r had significantly negative correlations (*P* < 0.01). In the near-infrared region of the spectra, hyperspectral reflectance dropped sharply with increasing LNA. There was a maximum correlation between LNA and raw hyperspectral reflectance at 790.4 nm (*r* = −0.874, Figure 1). In this study, LNA was set as dependent variable whereas hyperspectral reflectance was set as independent variable at a wavelength of 790.4 nm. To explore the relationship between LNA and hyperspectral reflectance, we established a model by using linear and non-linear fitting equations (exponential, logarithmic, parabolic, power, and cubic regressions). It can be seen that linear model and parabolic model fitted better with *R*^2^ values of 0.763 and 0.788, respectively. It was feasible to diagnose wheat LNA with these models. Furthermore, diagnosis accuracy needs to be evaluated. A simple linear model was selected because the goodness of fit was close for these two models.

### Correlation analysis and diagnostic model of wheat LNA with first-order differential hyperspectral reflectance

Equation (4) was used to define first-order differential spectra:

(4) $$\begin{array}{l} \rho^{\prime }\left(\lambda \right)=\frac{d\rho \left(\lambda \right)}{d\lambda } \end{array}$$

Equation (5) was used to calculate discrete spectral data:

(5) $$\begin{array}{l} \rho^{\prime }\left(\lambda \right)=\left[\rho \left(\lambda_{i+1}\right)-\rho \left(\lambda_{i-1}\right)\right]/\left(\lambda_{i+1}-\lambda_{i-1}\right) \end{array}$$

Herein, ρ(λ) and ρ′(λ) represented raw hyperspectral reflectance and first-order differential hyperspectral reflectance at wavelength λ, respectively.

**Figure 2:**
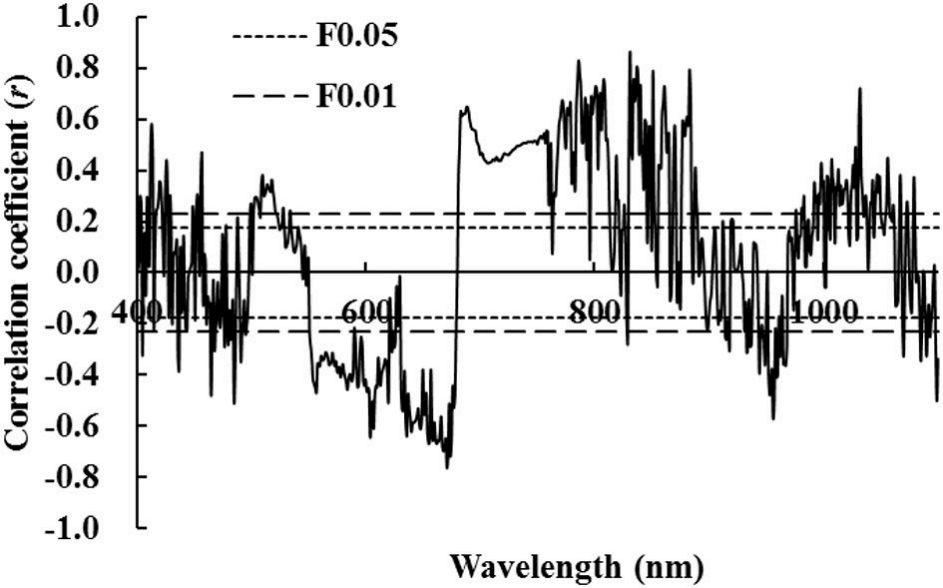
Correlation coefficient between first-order hyperspectral reflectance and wheat LNA (The correlation coefficient outside the two P_0.05_ lines was significant at 0.05 level, while the correlation coefficient outside the two P_0.01_ lines was significant at 0.01 level).

Figure 2 illustrates that there was no significant difference between correlation coefficients of LNA and first-order differential hyperspectral reflectance of wheat. Correlation coefficients were either completely significant or extremely significant in most spectral ranges. This indicates that LNA had a strong correlation with first-order differential hyperspectral reflectance. This was attributed to the strong absorption of chlorophyll in the visible region of electromagnetic spectrum. In the near infrared region of electromagnetic spectrum, it was primarily due to the influence of high frequency noises on differential spectra. Moreover, reflection and scattering interactions were observed between chlorophyll and internal structure of leaves. An extremely positive correlation was observed at a wavelength of 831.7 nm; a maximal coefficient of *r* = 0.864 (*P* < 0.01, Figure 2) was observed in this study. In this study, LNA was set as the dependent variable and first order differential hyperspectral reflectance [It was observed at a wavelength of 831.7 nm (ρ′_831.7_)] was set as the independent variable. The model was established by linear and non-linear fitting, including exponential, logarithmic, parabolic, power, and cubic regressions. We found that linear and exponential models fitted better with *R*^2^ values of 0.792 and 0.824, respectively. This indicates that exponential model was more suitable than linear model. By comparing aforementioned models, it could be inferred that first-order differential hyperspectral reflectance showed a better goodness of fit at a wavelength of 831.7 nm. This was better than that of raw hyperspectral reflectance at a wavelength of 790.4 nm (ρ_790.4_), regardless of linear or non-linear fitting. This indicates that it would be better to diagnose LNA with ρ′_831.7_; however, its diagnosis accuracy had to be further evaluated.

### Correlation analysis between wheat LNA and various hyperspectral characteristic parameters

Table 3 shows that nine characteristic variables were based on hyperspectral position, except for first-order maximal derivative inside red edge *(D*_*b*_*)* and corresponding band length with *D*_*b*_ *(*λ_*b*_*)* and wavelength with respect to *D*_*y*_ *(*λ*y)*. Table 3 also shows that there was no significant correlation with wheat LNA. The remaining six characteristic variables had a significant or extremely significant correlation with wheat LNA. Among them, the correlation between maximal first-order differential value occurred within the red edge (*D*_*r*_) and LNA was highest with *r* = −0.893. The three characteristic variables were analyzed from hyperspectral area. This indicates that the sum of first-order differentials was not significantly correlated within yellow edge (*SD*_*y*_) and nitrogen accumulation. An extremely significant positive correlation was observed between the sum of first-order differentials within the red edge (*SD*_*r*_) and LNA (*r* = 0.911, *P* < 0.01). An extremely significant negative correlation was observed between the maximum first-order differential spectra for blue edge (*SD*_*b*_) and LNA (*r* = −0.821, *P* < 0.01). Among the six variables related to vegetation indices, there was no significant correlation between following parameters: *R*_*g*_*/R*_*o*_ or *(R*_*g*_ –* R*_*o*_*)/(R*_*g*_ + *R*_*o*_*)* and LNA. Moreover, a significant positive correlation was observed between *SD*_*r*_*/SD*_*b*_ or *SD*_*r*_*/SD*_*y*_ and LNA (*P* < 0.05). An extremely significant positive correlation was observed between *(SD*_*r*_ – * SD*_*y*_*)/(SD*_*r*_ + *SD*_*y*_*)* or *(SD*_*r*_ − *SD*_*b*_*)/(SD*_*r*_ + *SD*_*b*_*)* and LNA. The *(SD*_*r*_ − *SD*_*b*_*)/(SD*_*r*_ + *SD*_*b*_*)*, hereinafter referred to as the NREAI (Normalized Red Edge Area Index), had the biggest correlation coefficient (*r* = 0.941, *P* < 0.01). Hyperspectral characteristic variables were identified for the diagnosis of wheat LNA. The optimal result was NREAI.

**Table 3:** Correlation coefficients between hyperspectral characteristic variables and wheat LNA (*n* = 144).

**Hyperspectral characteristic variable**	***r***
*D*_b_	−0.014
λ_b_	0.031
*D*_r_	−0.893^**^
λ_r_	0.820^**^
*D*_y_	0.843^**^
λ_y_	−0.009
*R*_g_	−0.186^*^
λ_g_	−0.194^*^
*R*_o_	−0.143^*^
*SD*_r_	0.911^**^
*SD*_b_	−0.821^**^
*SD*_y_	−0.146
*SD*_r_*/SD*_b_	−0.214^*^
*SD*_r_*/SD*_y_	0.201^*^
*R*_g_*/R*_o_	0.151
*(SD*_r_*−SD*_b_*)/(SD*_r_*+SD*_b_*)*	0.941^**^
*(SD*_r_*−SD*_y_*)/(SD*_r_*+SD*_y_*)*	0.856^**^
*(R*_g_*−R*_o_*)/(R*_g_*+R*_o_*)*	−0.137

* *P < 0.05, | r | > 0.174*;

** *P < 0.01, | r | > 0.228. The same below*.

### Linear and non-linear fitting models

To diagnose agronomic parameters more accurately, hyperspectral characteristic variables were selected as independent variables. These variables indicate that there was an extremely significant correlation with LNA. Furthermore, LNA was set as the dependent variable to analyze the correlation between hyperspectral characteristic variables (x) and LNA (y). Diagnostic models were constructed with following six functions: (1) Linear function: y = a + bx; (2) Exponential function: y = a × exp(bx); (3) Logarithm function: y = a + b × lnx; (4) Parabolic function: y = a + bx + cx^2^; (5) Power function: y = a × x^b^; and (6) Cubic function: y = a + bx + cx^2^ + dx^3^. Herein, y was the diagnostic value of LNA; x was the characteristic variable; and a, b, c, and d were the undetermined coefficients (constants) of diagnostic models.

Table 3 shows that most of the 18 hyperspectral characteristic variables had a significant or extremely significant correlation with LNA. Hyperspectral characteristic variables had an extremely significant correlation with LNA, so they were selected as candidate parameters. Linear and non-linear regression analysis was carried out. Then, fitting equations were established to determine the parameters suitable for hyperspectral diagnostic model of LNA (Table 4). It was observed that *R*^2^ values of the exponential diagnostic models were based on D_r_, λ_r_, and D_y_; moreover, maximal values were used to diagnose wheat LNA. Cubic diagnostic models were based on *SD*_*r*_ and *SD*_*b*_, which are defined as the highest *R*^2^-values. Furthermore, *R*^2^-values of models was represented by the equation NREAI, which was based on vegetation indices. All these values were higher than other hyperspectral characteristic variables, which were included in the same model types. The fitted results were obtained by constructed linear model; these results were very close to non-linear models. The mathematical expression of linear model was very simple; therefore, hyperspectral diagnostic model of LNA was used as the dependent variable (y). Furthermore, NREAI was defined as the independent variable (x). The relationship between these variables was expressed as follows: *y* = 553.472+738.822x, with *R*^2^ = 0.886.

**Table 4:** Linear and non-linear regression analysis between hyperspectral characteristic variables and wheat LNA (*n* = 144).

**Hyperspectral characteristic variable**	**Model**	**Model parameter**				***R^2^***
		**a**	**b**	**c**	**d**	
*D*_r_	Linear	23.346	−11.372			0.797^**^
	Exponential	11.037	−19.836			0.846^**^
	Logarithm	21.821	−88.931			0.803^**^
	Parabolic	39.024	−1021.93	−1674.36		0.839^**^
	Power	6.946	−12.877			0.816^**^
	Cubic	1121.84	−3452.92	4481.62	−109.74	0.819^**^
λ_r_	Linear	4022.63	599.37			0.673^**^
	Exponential	36.933	301.77			0.686^**^
	Logarithm	3.936	9.758			0.611^**^
	Parabolic	33.811	2.976	6.938		0.681^**^
	Power	34.733	6.292			0.637^**^
	Cubic	41.767	856.091	46.711		0.649^**^
*D*_y_	Linear	22.734	881.463			0.711^**^
	Exponential	32.071	14.877			0.802^**^
	Logarithm	73.791	263.88			0.643^**^
	Parabolic	542.766	3671.65	3722.62		0.791^**^
	Power	7.821	46.943			0.677^**^
	Cubic	12.833	48.773	83.552	−101.72	0.756^**^
*(SD*_r_ −*SD*_b_*)/(SD*_r_ + *SD*_b_*)*	Linear	553.472	738.822			0.886^**^
	Exponential	2116.82	4231.63			0.862^**^
	Logarithm	186.52	1863.81			0.872^**^
	Parabolic	92.573	7.736	−37.282		0.891^**^
	Power	291.778	101.627			0.862^**^
	Cubic	57.116	1928.93	−311.261	97.246	0.898^**^
*(SD*_r_ −*SD*_y_*)/(SD*_r_ + *SD*_y_*)*	Linear	43.623	13.383			0.733^**^
	Exponential	61.782	21.822			0.636^**^
	Logarithm	9.793	23.161			0.672^**^
	Parabolic	4.683	21.981	−8.572		0.696^**^
	Power	28.861	31.173			0.708^**^
	Cubic	13.711	33.908	−18.167	9.678	0.684^**^
*SD*_r_	Linear	11.127	42.657			0.829^**^
	Exponential	12.658	17.267			0.841^**^
	Logarithm	9.486	15.167			0.792^**^
	Parabolic	12.386	34.232	−12.186		0.841^**^
	Power	16.563	15.677			0.687^**^
	Cubic	6.637	16.574	−11.232	3.761	0.846^**^
*SD*_b_	Linear	13.346	−11.783			0.674^**^
	Exponential	8.774	−23.063			0.472^**^
	Logarithm	14.833	−6.843			0.562^**^
	Parabolic	5.927	−12.126	−2.103		0.683^**^
	Power	3.218	−9.253			0.617^**^
	Cubic	9.145	−6.264	−7.691	3.588	0.737^**^

*Linear function: y = a + bx; Exponential function: y = a × exp (bx); Logarithm function: y = a + b × lnx; Parabolic function: y = a + bx + cx^2^; Power function: y = a × b; Cubic function: y = a + bx + cx^2^ + dx^3^ (y was the diagnostic value of LNA; x was the characteristic variable; and a, b, c and d were the undetermined coefficients (constants) of diagnostic models.)*.

### Predictive test of models

Rajendran et al. (2009) proposed that “over-fitting” phenomenon was most likely to occur during model establishment stage. In most cases, when the number of samples was less than hyperspectral band number, hyperspectral reflectance could have no relationships with some physical and chemical components in crop, but noise must be related. A hyperspectral model was constructed on various physical and chemical parameters; the main objective was to construct a more reliable model that controls and eliminates over-fitting error.

**Figure 3:**
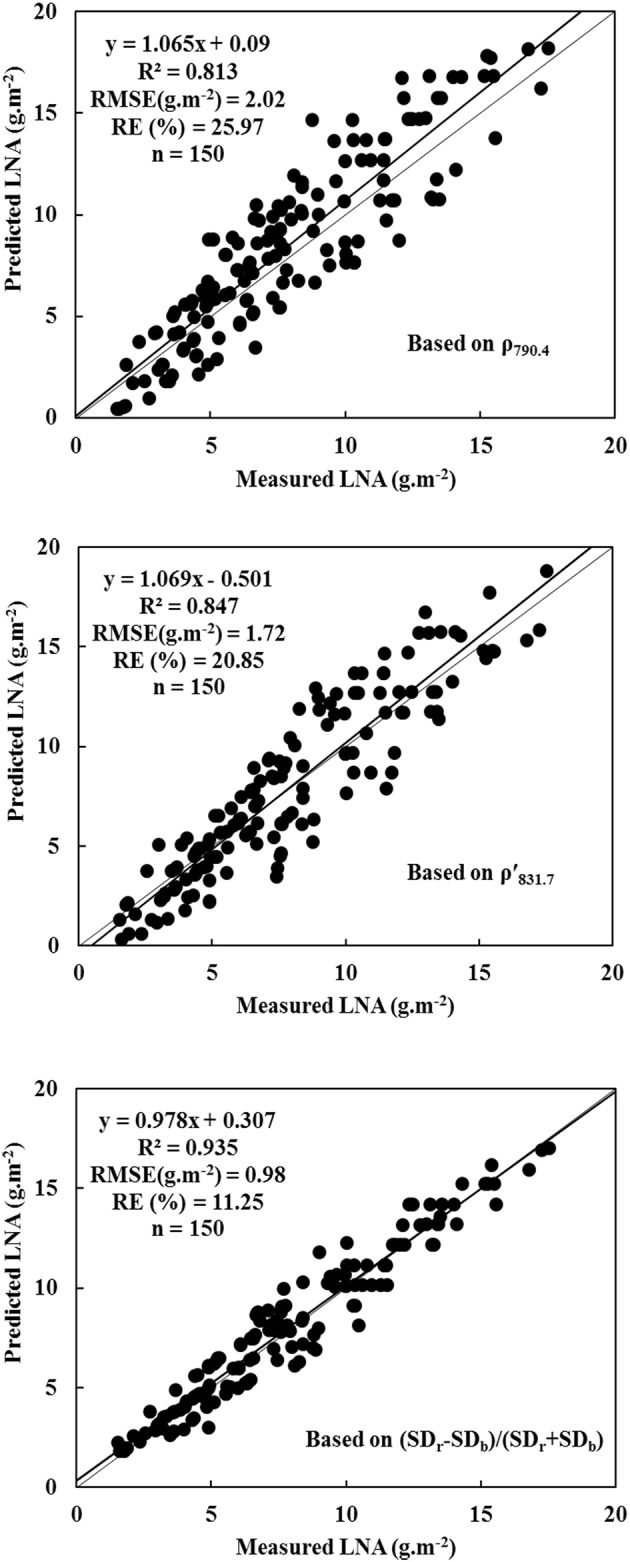
Evaluating the diagnostic models for wheat LNA based on ρ_790.4_, ρ′_831.7_ and (*SD*_*r*_ − *SD*_*b*_)/(*SD*_*r*_ + *SD*_*b*_) (model based on ρ_790.4_: *y* = 1.065x + 0.09, *R*^2^ = 0.813, RMSE (g·m^−2^) = 2.02, RE (%) = 25.97, *n* = 150; model based on ρ′_831.7_: y = 1.069x – 0.501, *R*^2^ = 0.847, RMSE (g·m^−2^) = 1.72, RE (%) = 20.85, *n* = 150; model based on (*SD*_*r*_ − *SD*_*b*_)/(*SD*_*r*_ + *SD*_*b*_): y = 0.978x + 0.307, *R*^2^ = 0.935, RMSE (g·m^−2^) = 0.98, RE (%) = 11.25, *n* = 150).

A novel regression equation of high significance was used to construct an optimal diagnostic model. This diagnostic model was a mathematical expression that minimized RMSE and RE. Thus, a reliable model was constructed with 2015 data. Figure 3 illustrates that diagnostic models were used to determine wheat LNA. The analysis was performed with respect to following parameters: ρ_790.4_, ρ′_831.7_, and NREAI. The normalized variable NREAI was considered as the independent variable to construct hyperspectral model of LNA. In this case, the largest *R*^2^-values were used along with the smallest RMSE and RE. The results indicate that hyperspectral characteristic variable of NREAI was the most appropriate diagnostic predictor of wheat LNA; *R*^2^, RMSE, and RE values were 0.935, 0.98 g·m^−2^, and 11.25%, respectively. Therefore, *y* = 553.472+738.822 NREAI was the diagnostic model of wheat LNA. Compared to the diagnostic model based on NREAI, the two diagnostic models based on ρ_790.4_ and ρ′_831.7_ showed slightly poor performance as they could not reach saturation levels. These diagnostic models were used to diagnose higher LNA level. When diagnostic model was used to process 2016 data, it was found that ρ_790.4_ and ρ′_831.7_ were closely related to wheat LNA; however, their diagnosis ability was increased to a higher level. Thus, the two hyperspectral parameters of ρ_790.4_ and ρ′_831.7_ were also considered as potential indicators in the diagnosis of wheat LNA.

**Figure 4:**
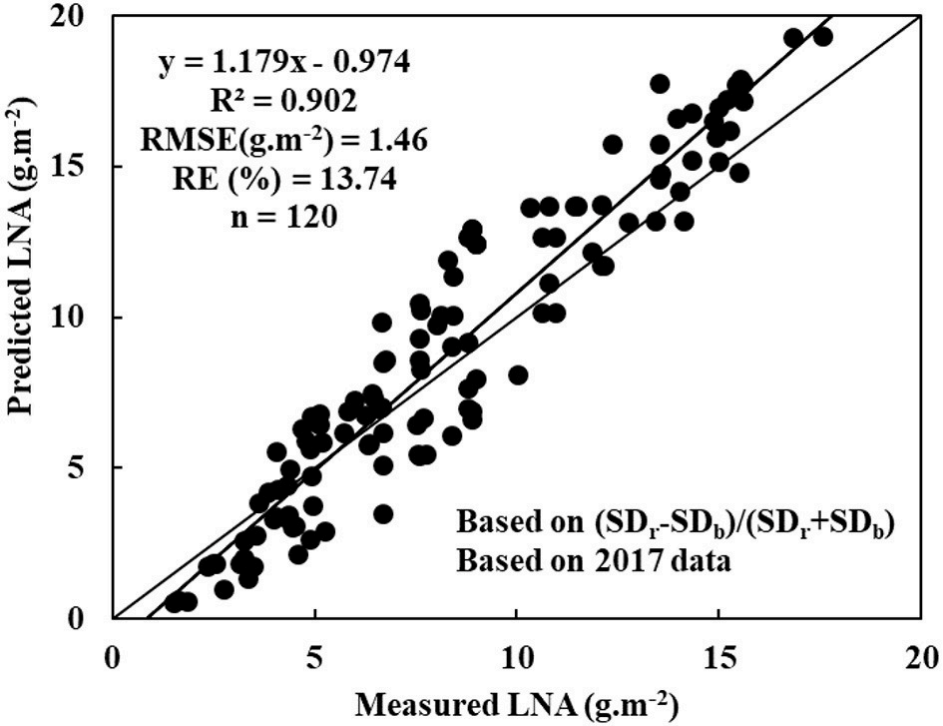
Evaluating the diagnostic model based on (SD_r_−SD_b_)/(SD_r_+SD_b_) using a different cultivar or even wheat grown elsewhere: *y* = 1.179x - 0.974, *R*^2^ = 0.902, RMSE (mg.g^−1^) = 1.46, RE(%) = 13.74, *n* = 120).

The experimental data was further tested and evaluated in different time and space conditions to display the reliability and universality of diagnostic model. Here, we analyzed 120 samples that were obtained in 2017 data. Thses samples were obtained from a different cultivar or even wheat grown elsewhere. The data was used to test LNA model, which was based on NREAI. Figure 4 shows that the model was obtained with *R*^2^, RMSE, and RE values of 0.902, 1.46 g·m^−2^, and 13.74%, respectively. Therefore, it was not only feasible that the NREAI was selected to diagnose wheat LNA, but also was possible to be applied widely in future.

## Discussion

Leaf is the key site for photosynthesis. Nitrogen concentration depends on the formation of chlorophyll in leaves. Chlorophyll plays an important role in the formation and transportation of organic compounds. In this experiment, LNA, LNC, and unit leaf dry weight was more effectively reflected by nitrogen status of wheat. These parameters were used to characterize nutritional status of wheat canopy, which indicated the potential of wheat production.

Hyperspectral data has been extensively used to diagnose LNC of crops (Daniela et al., 2009; Vigneau et al., 2011; Knyazikhin et al., 2013). However, a generalized diagnostic model was not used due to its numerous influencing factors (Morón et al., 2007; Inoue et al., 2012; Miphokasap et al., 2012). In previous studies, different varieties of wheat were investigated with various hyperspectral parameters; however, they still lacked transformation characteristics. We proposed a normalized variable NREAI, which was based on vegetation indices. This provided a basis for non-destructive monitoring and accurate diagnosis of wheat LNA. Very few studies have investigated different components of wheat with a variety of hyperspectral parameters. There are very few transformation forms. In this study, a normalized variable NREAI, which was based on vegetation indices, was proposed to sensitively diagnose wheat LNA. This provides a basis for non-destructive monitoring and accurate diagnosis of wheat LNA. Besides, we investigated the existing theory and technology associated with hyperspectral diagnosis of wheat LNA.

Scientists have conventionally performed a correlation analysis between wheat LNA and raw hyperspectral reflectance for determining nitrogen levels at different stages (Ryu et al., 2011; Muharam et al., 2015). First-order differential hyperspectra, hyperspectral characteristic variables, and all kinds of vegetation indices have shown that nitrogen accumulated mostly in leaves. In near infrared region of the spectra, hyperspectral reflectance showed a positive correlation with nitrogen accumulation; however, hyperspectral reflectance showed a negative correlation with visible light spectra. The results agreed completely with research results of maize, which was determined in a previous study conducted by Alchanatis et al. (2005). In a linear regression model, LNA was determined in wheat by using ρ_790.4_ as an independent variable. In an exponential model, LNA was diagnosed in wheat by using ρ′_831.7_ as the independent variable. The results agreed completely with the linear model. In fact, the exponential model produced much better results than the linear model. Using six types of regression functions, we discovered that wheat LNA could be diagnosed with following hyperspectral parameters: hyperspectral position, hyperspectral area, and vegetation indices. The normalized variable NREAI was considered as an independent variable for statistically determining three evaluation criteria. We constructed hyperspectral diagnostic model of wheat LNA. Wheat LNA was determined from the following expression: *y* = 553.472 + 738.822 NREAI. Furthermore, the LNA model based on NREAI was useful in the application of a different cultivar or even another growth region. However, the mechanisms of results should be studied further to determine whether this model was significantly affected by more wheat cultivars or growth regions.

The goodness of LNA fit, which was obtained with respect to ρ′_831.7_ (whether linear or non-linear), was found to be higher than that obtained with respect to ρ_790.4_. This agreed completely with the results of a study conducted by Fitzgerald et al. (2010) Nevertheless, this study did not consider hyperspectral differences of several wheat varieties. Further studies were conducted to determine whether significant differences in wheat cultivars had an impact on this model and mechanisms of results. The conclusions of our study were based on the data collected from two sites in three years. In addition, there were few cultivars, limited number of samples, and limited range of measured wavelengths. Moreover, no intensive measurements were performed during different growth periods. Therefore, further studies must be conducted to determine whether optimal hyperspectral parameters could be used to estimate the stability and accuracy of predictive results with respect to wheat LNA. In the near future, studies must also investigate whether the proposed diagnostic model was suitable for determining LNA of more wheat cultivars in large areas.

The present study investigated and evaluated the relationships between following parameters: LNA and raw hyperspectral reflectance, first-order differential hyperspectral reflectance, and hyperspectral characteristics of wheat. Wheat LNA could also be diagnosed with hyperspectral data. To diagnose wheat LNA, raw hyperspectral reflectance (measurement wavelength = 790.4 nm) and first-order differential hyperspectral reflectance (measurement wavelength = 831.7 nm) were considered as optimal techniques. Nevertheless, the better one was the normalized variable NREAI. This indicates a superior fit and better performance. Moreover, NREAI was more effective in diagnosing LNA than raw hyperspectral reflectance, first-order hyperspectral reflectance, and red-edge parameters. It might also explain why different hyperspectral variables must be used to diagnose crop LNA. In totality, NREAI would be considered as a new predictor of wheat LNA in future.

## Author contributions

CT and WG conceived the research. CT, JZ, and YD designed and performed the experiments. CT and YD prepared and revised the manuscript. CT, ML, and DW analyzed the data. ML, DW, and YZ provided technical. All authors discussed the results and commented on the manuscript.

### Conflict of interest statement

The authors declare that the research was conducted in the absence of any commercial or financial relationships that could be construed as a potential conflict of interest.
